# Biomechanical stress unmasks a fibroblast-dependent hypercontractile-disarray phenotype in MYBPC3 truncation HCM

**DOI:** 10.1063/5.0336866

**Published:** 2026-09-14

**Authors:** Huanzhu Jiang, Ganesh Malayath, Nongmaithem Debeni Devi, Hsin-Yi Cindy Chou, Yasaman Kargar Gaz Kooh, Jingxuan Guo, Ghiska Ramahdita, Jenna Nguyen, Riya Bhakta, Lavanya Aryan, Sharon Cresci, Nathaniel Huebsch

**Affiliations:** 1 Department of Biomedical Engineering, Washington University School of Engineering, St. Louis, Missouri 63130, USA; 2 The Institute of Materials Science & Engineering, Washington University School of Engineering, St. Louis, Missouri 63130, USA; 3 Department of Mechanical Engineering & Materials Science, Washington University School of Engineering, St. Louis, Missouri 63130, USA; 4 Cardiovascular Division, Department of Medicine, and Department of Genetics, Washington University School of Medicine, St. Louis, Missouri 63130, USA

## Abstract

Hypertrophic cardiomyopathy (HCM) is commonly caused by pathogenic variants in the sarcomere, such as truncations in myosin-binding protein C (MYBPC3), yet clinical severity and adverse outcomes correlate poorly with genotype alone and are strongly influenced by fibrosis. We developed an engineered human micro-heart tissue (*μ*HT) combining iPSC-derived cardiomyocytes (CM) with defined primary cardiac fibroblast (cFB) fractions and mechanically modulated afterload. This design allowed us to test how stromal context and pro-fibrotic signaling shape HCM pathogenesis in micro-engineered heart tissues made from iPSCs harboring an HCM-linked frameshift variant in *MYBPC3* (MYBPC3fs). Under low afterload, genotype-associated differences in total active force were limited at 0% and 5% added cFB but became apparent at 25% added cFB. However, high afterload markedly amplified MYBPC3fs *μ*HT hypercontractility, with tissues generating greater total active force at both 5% and 25% added cFB. We also observed afterload- and cFB-dependent remodeling of Ca^2+^ handling, indicating that excitation–contraction coupling in MYBPC3fs tissues is modulated by combinatorial actions of mechanical stress and the stromal environment. Despite heightened contractile output, the MYBPC3fs *μ*HT exhibited blunted improvements in Z-disc alignment with increasing cFB content, whereas cardiomyocytes within the isogenic control *μ*HT showed progressive structural ordering as cFB density increased. Finally, the MYBPC3fs *μ*HT exhibited a greater response to TGF-β, leading to exaggerated increases in tissue-level resting tension and α-smooth muscle actin expression. Our findings provide important insights into the pathophysiologic pathways leading to adverse outcomes in clinical HCM and a foundation for future studies of cardiomyocyte–fibroblast interactions in HCM.

## INTRODUCTION

Hypertrophic cardiomyopathy (HCM) is the most common inherited cardiac disease and is characterized by left ventricular hypertrophy in the absence of another cardiac, systemic, or metabolic disease capable of producing the magnitude of hypertrophy in a given patient.^1–3^ Individuals with HCM may have adverse clinical outcomes, including heart failure, arrhythmias, and sudden cardiac death (SCD).^1,4^ The prevalence of HCM is thought to be between 1:500 and 1:200, underscoring the fact that HCM may be more common than previously appreciated.^5–7^

HCM is most commonly caused by pathogenic variants in sarcomeric genes expressed in cardiomyocytes (CMs), and loss-of-function variants in *MYBPC3*, which encodes cardiac myosin-binding protein C (MYBPC) in cardiomyocytes, represent one of the most common genetic causes of HCM (roughly 40% of cases).^2,8^ Mechanistically, MYBPC regulates thick-filament behavior and cross-bridge recruitment.^8–11^ MYBPC3 truncation/haploinsufficiency can shift myosin toward more contractile states, leading to hypercontractility and energetic inefficiency.^8,12–15^ Consistent with this, cardiomyocytes isolated from HCM patients harboring *MYBPC3* variants exhibited energetic deficiency of contraction (higher ATPase activity during paced contractions).^16^

A critical challenge in managing patients with HCM is that the clinical onset, progression, and severity of this disease are highly variable.^17^ Variable expression has been observed even in relatives carrying the same pathogenic or likely pathogenic variant and between identical twins.^18,19^ This variability has driven extensive use of iPSC-based^20–29^ and animal models^30–32^ to dissect how genetic and non-genetic factors interact to shape HCM progression. These models are valuable because myocardial tissue from healthy, pathogenic/likely pathogenic-positive, HCM-phenotype-negative individuals is not practically obtainable. Findings from these models highlight that genotype alone does not fully explain HCM severity or clinical outcomes.^33^ Instead, disease progression is strongly influenced by non-genetic triggers, notably including the degree of myocardial fibrosis and environmental mechanics (blood pressure).^3,17,20,30,34–36^

Prior evidence from clinical studies^37–42^ strongly suggests that cardiac fibroblasts (cFBs) play a role in HCM pathogenesis. Myocardial fibrosis is an early hallmark and a major modifier of HCM progression and outcomes and is associated with changes in tissue mechanics.^42–45^ In the healthy heart, cFBs play a central role in shaping myocardial structure and function through extracellular matrix (ECM) deposition, paracrine signaling, and electrical coupling with cardiomyocytes.^46–48^ Under pathological conditions (e.g., with pathological mechanical stress or chemical cues like transforming growth factor-beta, TGF-β), fibroblasts differentiate into α-smooth muscle actin (α-SMA)-positive myofibroblasts.^49–52^ Fibroblasts and/or myofibroblasts influence cardiomyocyte biology through both chemical and mechanical means.^53^ For example, myofibroblast activation increases ECM deposition and resting (diastolic) tension.^54,55^ Together, these changes can elevate tissue stiffness.^55^ In turn, altered mechanics and fibroblast-cardiomyocyte coupling can disrupt electrical stability and excitation–contraction coupling,^47,56,57^ promoting maladaptive remodeling. In addition, mechanical cues alone could further accelerate pathologic fibroblast/cardiomyocyte crosstalk because pathological mechanical feedback (from a stiffer environment) can further amplify profibrotic signaling in fibroblasts.^56,58,59^ For example, myofibroblasts' tensile forces can activate latent TGF-β stored in the ECM, creating a self-reinforcing mechano-TGF-β loop.^60^ Beyond matrix mechanics, cFBs exert direct chemical influences on cardiomyocyte function. Fibroblasts secrete growth factors, cytokines, and vasoactive mediators (including TGF-β family signaling, interleukins, endothelin-1, and ECM-derived matrikines) that can modulate cardiomyocyte hypertrophic signaling, calcium handling, electrophysiology, and stress responses.^55,56,61–63^ Additionally, fibroblast–cardiomyocyte electrical coupling and paracrine–electrotonic interactions can alter impulse propagation and repolarization stability, linking stromal remodeling to arrhythmia susceptibility.^62,64–66^ Recent evidence further supports variant-dependent cardiomyocyte-to-stromal crosstalk: HCM-associated pathogenic variants can drive stromal activation through EGFR-mediated paracrine signaling pathways, underscoring a mechanistic axis by which sarcomere dysfunction may promote fibrosis and remodeling.^23^ Together, these findings support a model in which genetics establishes a sensitivity baseline, while mechanics and stromal signaling determine whether tissues remain compensated or transition to maladaptive remodeling. Importantly, TGF-β can signal directly in both cardiac fibroblasts and cardiomyocytes.^67,68^ Consequently, tissue-level responses to exogenous TGF-β cannot be assumed to arise exclusively from fibroblast activation.

The facts that (1) HCM-associated variants are found in proteins like MYBPC3 that are unique to cardiomyocytes^2,4,8^ and (2) fibrosis is a known regulator of HCM progression and outcomes^30,41,42^ strongly suggest that crosstalk between cFBs and CMs plays a role in the pathogenesis of HCM. CMs detect mechanical stress through sarcomeric and cytoskeletal structures, including integrin-costamere complexes and stretch-activated ion channels, which convert mechanical strain into biochemical signals that regulate contractility, calcium handling, and hypertrophic gene programs.^69,70^ cFBs are similarly mechanosensitive cells that respond to increased matrix stiffness and mechanical strain by activating profibrotic signaling pathways and differentiating into contractile α-SMA-positive myofibroblasts.^52,71,72^ As both cell types are directly affected by mechanical cues,^52,56,58,72^ it is reasonable to expect that mechanical cues affect this crosstalk. However, the quantitative relationship linking mechanical cues (e.g., afterload) and cFB fractions to the development of HCM pathology is not established. Moreover, while profibrotic cues like TGF-β are strongly implicated in myocardial remodeling,^32^ it remains unclear whether they merely suppress function uniformly or instead reprogram load sensitivity in a genotype- and composition-dependent manner.

Here, we developed an engineered human micro-heart tissue (*μ*HT) model integrating iPSC-derived cardiomyocytes (iPSC-CMs), defined added fractions of primary human cFBs, and controlled mechanical afterload to investigate how cardiomyocytes with an HCM patient-associated *MYBPC3* truncation variant (MYBPC3fs) respond to combinatorial changes in afterload and cFB presence. We formed *μ*HTs by co-encapsulating iPSC-CMs and primary cFBs into collagen-ECM-based gels within micro-wells surrounding poly(dimethylsiloxane) (PDMS)-based posts.^73,74^ We tuned afterload by manipulating PDMS post-bending stiffness and found that afterload acted together in a combinatorial fashion with the density of cFB to trigger genotype-dependent structural and functional tissue remodeling. This design enabled independent manipulation of mechanical load and added stromal-cell content while permitting measurement of active force, resting tension, Ca^2+^ transients, and tissue organization. We used the platform to determine how afterload and cFB content modify functional and structural phenotypes associated with an HCM-linked *MYBPC3* truncation variant. We additionally characterized the integrated response of these tissues to exogenous TGF-β as a tissue-level profibrotic remodeling stimulus. The model developed here will set the foundation for future studies dissecting important mechanisms for cFB/CM crosstalk in HCM, including analysis of how this crosstalk is manipulated by afterload, and may identify potential novel therapeutic targets for the treatment of HCM.

## RESULTS

### An engineered human micro-heart tissue (*μ*HT) platform enables independent control of fibroblast density and mechanical afterload

**FIG. 1.**
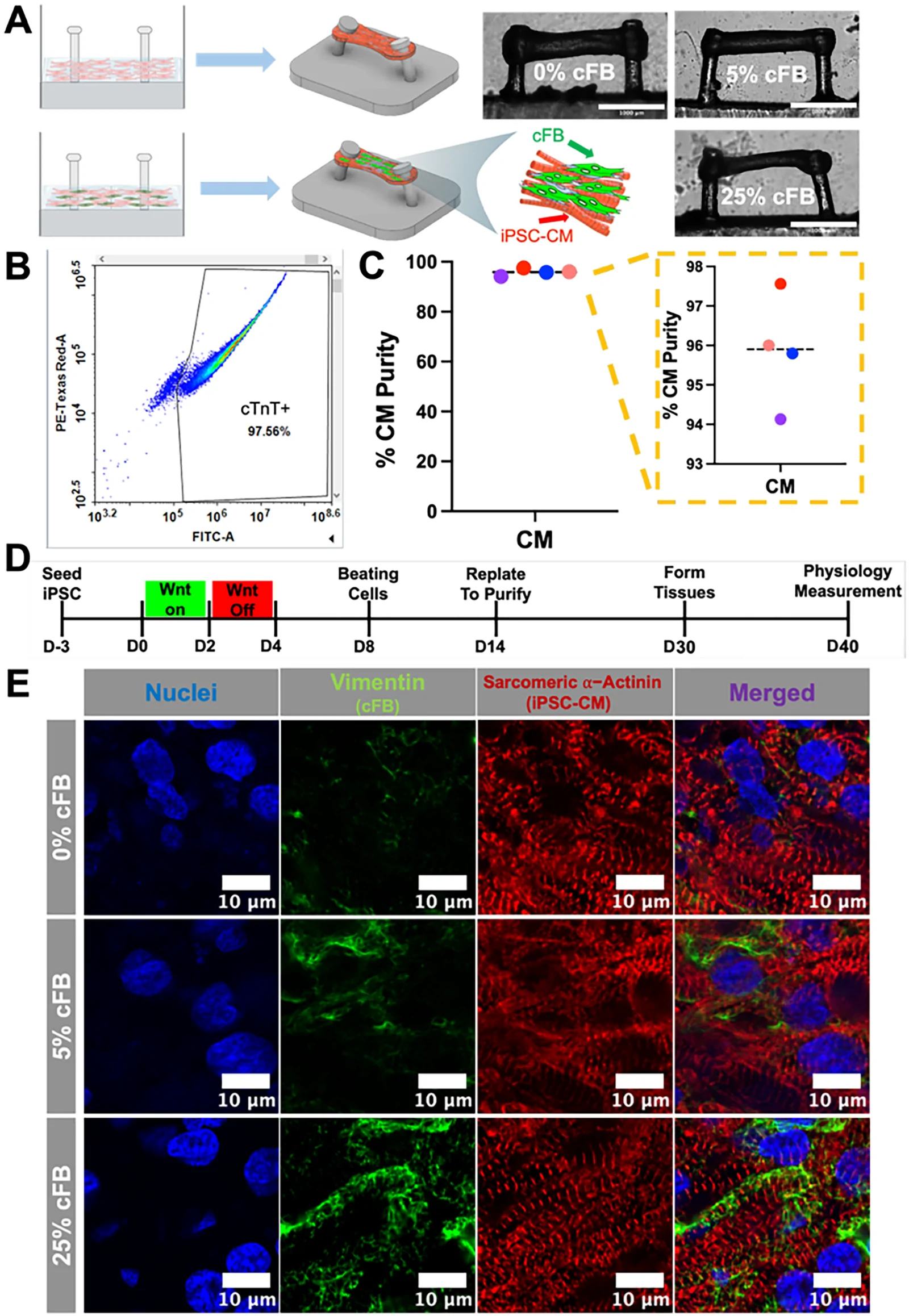
Generation of micro-heart tissues on two-post-based devices. (a) Schematic of the micro-heart tissue device design and side view of micro-heart tissues. Scale bar: 1000 *μ*m. (b) Representative graph of flow cytometry showing the percentage of cTnT-positive cells after 6 days of lactate purification. (c) The purity of cardiomyocytes after 6 days of lactate purification. (d) Timeline for hiPSC-CM production and tissue making. (e) Representative immunostaining confocal micrographs of isogenic control tissues containing 0%, 5%, and 25% cFB, respectively, stained for cFB (vimentin, green) and cardiomyocytes (α-actinin, red), and counterstained with the nuclear stain Hoechst (blue) on stiff posts.

To model the development of HCM, we first generated iPSCs harboring a patient-specific loss-of-function frameshift variant in *MYBPC3* (G1206fs;^75^ supplementary material, Fig. 1). In our prior studies, we modeled changes in afterload on the heart by forming iPSC-CM-based *μ*HT atop soft PDMS substrates.^24^ However, we found that adding even a modest fraction of cFB to these tissues led to consistent tissue delamination from the PDMS substrate [supplementary material, Fig. 2(a)]. This challenge has been reported in prior studies by other teams.^76^ This obstacle, along with the inability to monitor diastolic tension of the *μ*HT (a surrogate of fibroblast activation state^77^), prompted us to switch to the use of PDMS-post-based *μ*HT [Fig. 1(a)]. The post-bending stiffness was tuned between soft (0.42 N/m) and stiff (1.96 N/m) states by varying the composition of PDMS.^73^

To achieve robust *μ*HT formation with defined cell populations, we next optimized lactate media-based iPSC-CM purification to achieve high iPSC-CM purity and maintain iPSC-CM viability at a sufficiently high level to enable the purified cardiomyocytes to reproducibly form *μ*HTs across multiple differentiation batches. Six days of treatment in lactate-based media was most optimal as per these criteria and resulted in uniformly high iPSC-CM purity levels [Fig. 1(c)]. We formed *μ*HTs by combining this purified iPSC-CM fraction with 0%, 5%, or 25% additional primary cFBs, maintaining total cell density constant, and monitored *μ*HT phenotypes over time [Fig. 1(d)]. The 0%, 5%, and 25% conditions represent targeted fractions of primary cFBs added at tissue seeding, while the total input-cell number was held constant. This input range of cells reflects the composition of cFBs used in prior studies with engineered myocardium. At the lower end, 5% cFBs is used by some teams because in their studies, higher cFB fractions were associated with increased spontaneous and ectopic activity.^78^ However, several other teams report the construction of engineered myocardium with approximately 25%–30% cFBs. Because only these three unequally spaced added-cFB fractions were examined, these experiments were not designed to distinguish a continuously graded response from a nonlinear or threshold-dependent response—this would be an interesting topic for future study.

Immunostaining confirmed robust cardiomyocyte sarcomeric structure (sarcomeric α-actinin, staining of Z-discs) and the expected increase in vimentin-positive stromal cell signal with increasing cFB percentage, indicating successful assembly of multicellular tissues with controlled composition [Fig. 1(e)]. Gross tissue morphology and signal from the GCaMP6f genetic Ca^2+^ indicator within iPSC-CMs were consistent across *μ*HTs made from varying cFB fractions in representative brightfield and fluorescence images [supplementary material, Fig. 2(b)].

### MYBPC3 truncation confers load- and fibroblast-dependent hypercontractility that is most evident under high afterload

**FIG. 2.**
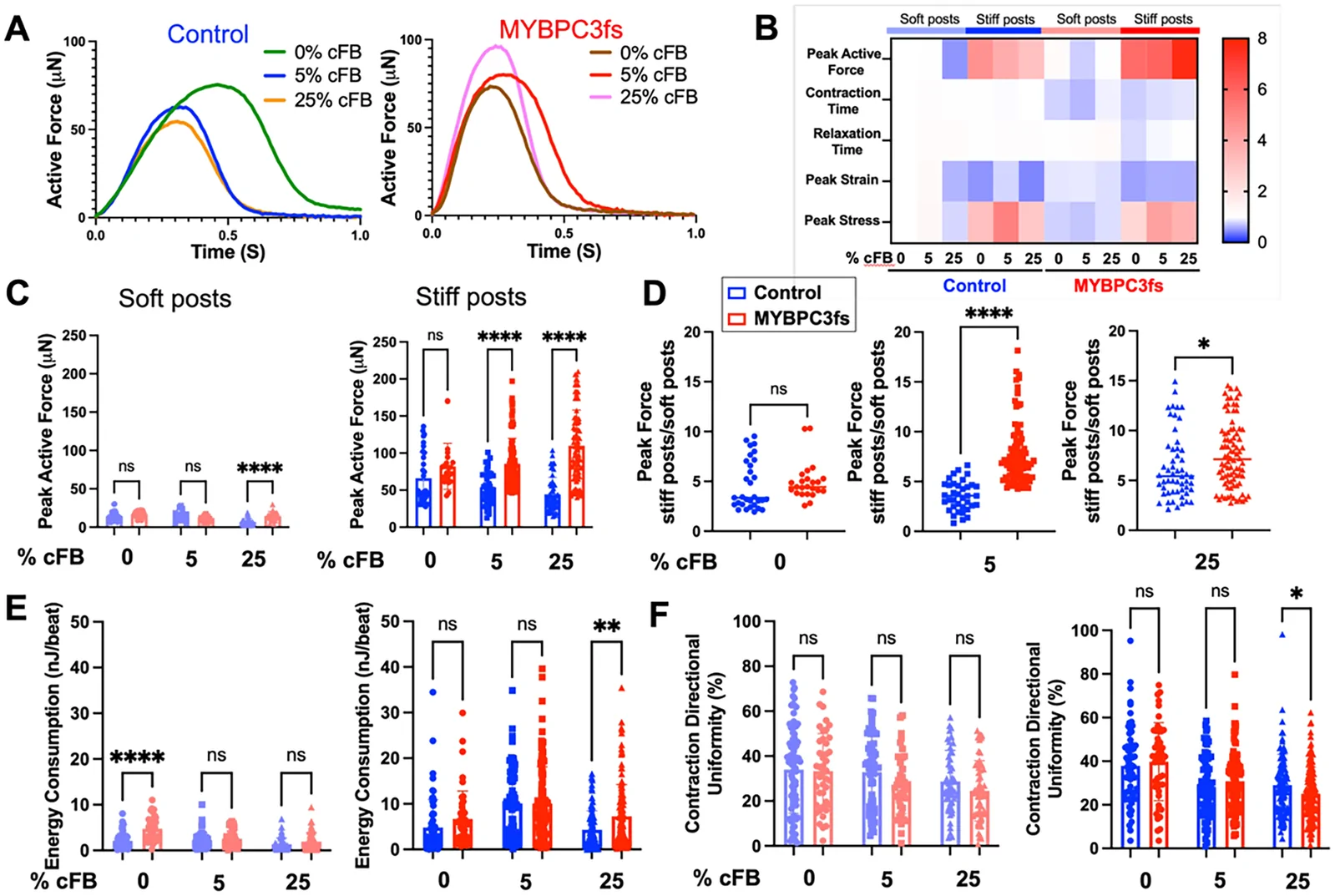
HCM mutant tissues containing a higher density of cFBs exhibit hypercontractile properties. (a) Representative force kinetics of control and mutant tissues with 0%, 5%, and 25% cFB on stiff posts. (b) Heatmap of force kinetics in control and mutant tissues associated with increasing post stiffness and cFB densities. All values were normalized to the mean value of the isogenic control tissues cultured on soft posts. (c) Active force of control and mutant tissues on soft (left) and stiff (right) posts. (d) Stiff post/soft post ratio across fibroblast fractions in control and mutant tissues. (e) Energy consumption of control and mutant tissues on soft posts (left) and stiff posts (right). (f) Contraction directional uniformity of control and mutant tissues on soft posts (left) and stiff posts (right). N values represent ≥3 independent batches. Error bar: *SD*. ∗, ∗∗, and ∗∗∗∗ indicate *p* value less than 0.05, 0.01, and 0.0001, respectively.

We next measured twitch force generation to quantify contractile mechanics (Fig. 2). Representative force traces of *μ*HTs working against stiff posts showed that increasing cFB fraction altered twitch waveform morphology in both genotypes, with a larger twitch amplitude in MYBPC3fs tissues at higher cFB fractions [Fig. 2(a), supplementary material, Fig. 3(a)]. Heatmap visualization of parameters related to contractile amplitude and kinetics confirmed a coordinated shift in mechanical performance, most evident under stiff-post loading of the MYBPC3fs mutant [Fig. 2(b)]. In *μ*HTs working against soft posts, genotype-driven differences in peak active force were modest at 0% and 5% cFB, but became pronounced at 25% cFB, where MYBPC3fs tissues generated ∼2-fold higher peak active force than controls [Fig. 2(c)]. In contrast, in *μ*HTs working against stiff posts, genotype-based separation was robust. MYBPC3fs tissues exhibited significantly higher peak active force than controls at 5% cFB (∼1.6-fold) and 25% cFB (∼2.5-fold), while differences at 0% cFB were not statistically significant [Fig. 2(c)].

Peak strain and stress were quantified in tissues [supplementary material, Figs. 3(e) and 3(f)]. Peak strain generally followed the force response because both measurements are derived from post displacement. Accordingly, under stiff-post loading, MYBPC3fs tissues containing 25% added cFB exhibited greater peak strain than corresponding control tissues [supplementary material, Fig. 3(e), right].

To determine whether tissue geometry contributed to the observed force differences, we incorporated measured tissue thickness into the cross-sectional area calculation [supplementary material, Figs. 5(e) and 5(f)]. Because side-view thickness measurements were available only for stiff-post tissues, the incorporation of tissue thickness in calculating the cross-sectional area and active-stress analyses was restricted to *μ*HTs working against stiff-posts. Tissue thickness was measured from relaxed-state side-view images and combined with individual top-view width measurements to estimate cross-sectional area using an elliptical approximation [supplementary material, Figs. 5(e) and 5(f)]. Measured tissue width did not differ significantly between genotypes in fibroblast-containing conditions [supplementary material, Fig. 3(d)]. In untreated stiff-post tissues, the thickness did not differ significantly between control and MYBPC3fs tissues at 0% or 5% added cFB [Fig. 6(k)]. At 25% added cFB, MYBPC3fs tissues were significantly thicker than controls [Fig. 6(k)].

After normalizing active force to the estimated width- and thickness-derived cross-sectional area, active stress did not differ significantly between genotypes at 0% or 25% added cFB and was lower in MYBPC3fs tissues at 5% added cFB [supplementary material, Fig. 3(f), right]. Thus, the greater total force generated by MYBPC3fs tissues was not accompanied by uniformly greater estimated active stress. In particular, the increased thickness observed at 25% added cFB contributed to attenuation of the genotype difference in stress after geometry normalization.

To directly test genotype-dependent sensitivity to afterload, we computed the ratio of peak twitch force in stiff/soft post-based *μ*HTs at each cFB density. Mutant tissues displayed a markedly elevated stiff/soft ratio at 5% cFB and a smaller but significant elevation at 25% cFB, whereas this “mechanical sensitivity ratio” was not different between genotypes when 0% cFB was added to the tissues [Fig. 2(d)]. Thus, while the active force increased in both genotypes to adapt to the increase in afterload, MYBPC3fs tissues exhibited a stronger mechanical sensitivity.

Beyond peak force, contraction kinetics and mechanical output metrics revealed genotype- and load-dependent functional remodeling. At almost every density of cFB in *μ*HTs working against posts of both stiffness levels, MYBPC3fs tissues exhibited faster contraction kinetics [supplementary material, Fig. 3(b)]. Relaxation time, by contrast, showed little genotype-driven separation on soft posts, but was significantly shorter in MYBPC3fs tissues at 0% and 5% cFB on stiff posts [supplementary material, Fig. 3(c)]. Together, these metrics reinforce that afterload and stromal context modulate not only peak force but also twitch timing and stress/strain output, with the most consistent changes in MYBPC3fs tissues emerging under stiff-post loading and high cFB density.

Consistent with remodeling of contractile function and past observations that pathogenic *MYBPC3* variants alter cardiomyocyte contractile energetics,^24^ estimated mechanical energy consumption per beat differed by genotype in a condition-dependent setting. On soft posts, MYBPC3fs tissues showed higher energy consumption than controls at 0% cFB, but no obvious genotype differences at 5% or 25% cFB fractions [Fig. 2(e), left]. In *μ*HTs working against stiff posts, calculated mechanical energy consumption diverged most strongly at 25% cFB [Fig. 2(e), right]. Notably, this energetic divergence coincided with reduced contraction directional uniformity in mutant tissues under the stiff-post, high-cFB condition [Fig. 2(f)]. Because post-deflection reports only the coordinated, axial component of contraction, reduced contraction directional uniformity suggests that a larger fraction of contractile work may be dissipated internally rather than contributing to axial post loading. In turn, this implies that the true energetic cost of contraction in MYBPC3fs tissues may be underestimated by post-deflection–based calculations, and thus the actual mechanical efficiency of contraction in these tissues is likely to be even lower than estimated by the direct energy consumption calculation.

### MYBPC3fs tissues display accelerated pacing and altered calcium remodeling influenced by afterload and fibroblast content

**FIG. 3.**
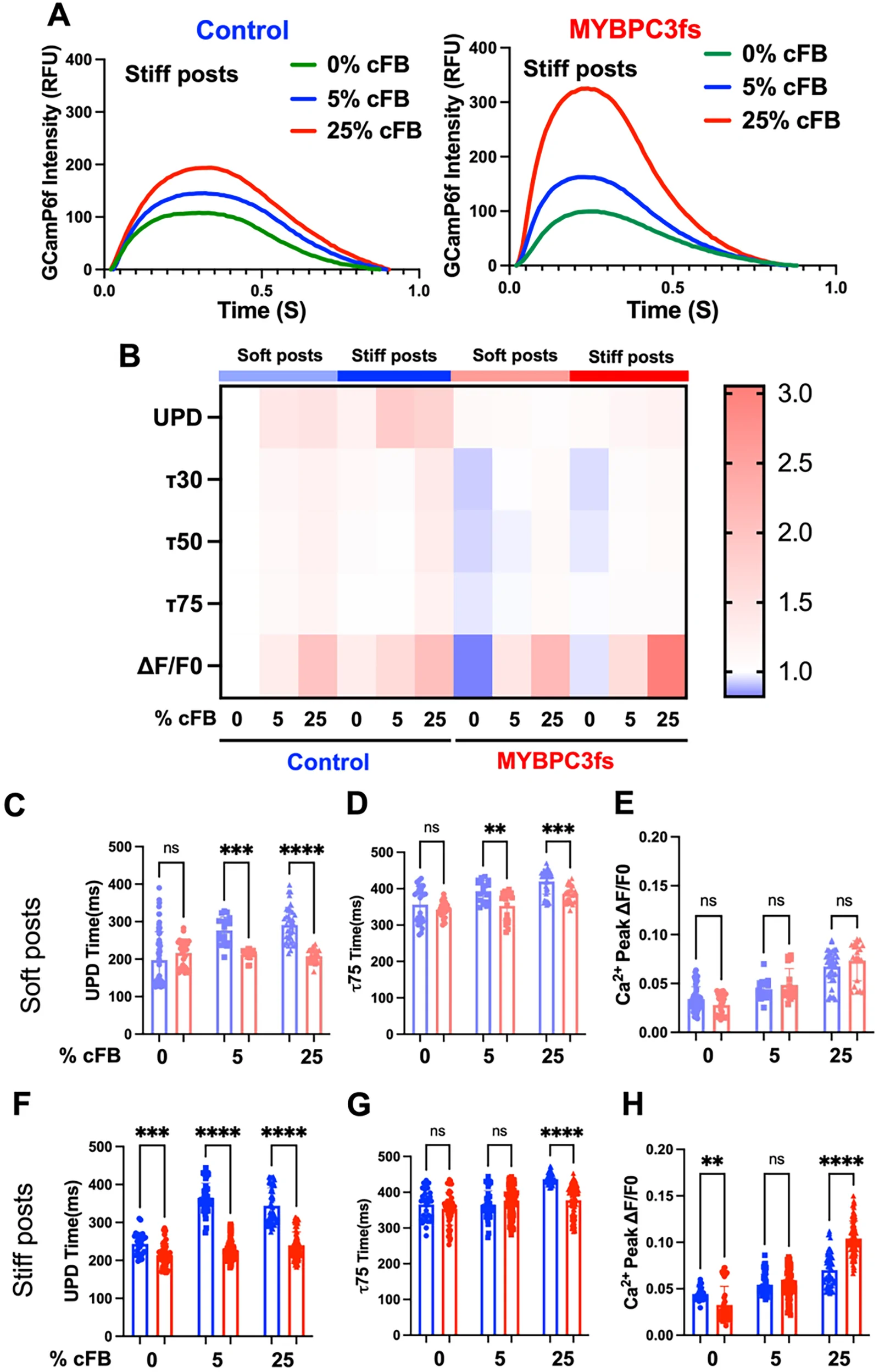
MYBPC3fs-deficient tissues express impaired calcium handling compared to controls. (a) Ca^2+^ transients of control tissues (left) and mutant tissues (right) with increasing cFB densities. (b) Heatmap of calcium handling in control and mutant tissues associated with increasing post stiffness and cFB densities. (c) Upstroke duration time of control and mutant tissues on soft posts. (d) Decay time of control and mutant tissues on soft posts. (e) Background-corrected peak Ca^2+^ intensity of control and mutant tissues on soft posts. (f) Upstroke duration time of control and mutant tissues on stiff posts. (g) Decay time of control and mutant tissues on stiff posts. (h) Background-corrected peak Ca^2+^ intensity of control and mutant tissues on stiff posts. N values represent ≥3 independent batches. Error bar: *SD*. ∗, ∗∗, ∗∗∗, and ∗∗∗∗ indicate *p* value less than 0.05, 0.01, 0.001, and 0.0001, respectively.

Because HCM phenotypes integrate contractile mechanics with excitation–contraction coupling, we next assessed cytoplasmic Ca^2+^ dynamics in the *μ*HTs through the endogenously expressed GcaMP6f indicator (Fig. 3). MYBPC3fs tissues exhibited higher spontaneous beating rates than controls across both post stiffnesses and across cFB fractions [supplementary material, Fig. 2(c)], consistent with an altered baseline electromechanical state. To avoid confounding calcium kinetics by differences in intrinsic pacing, all subsequent calcium analyses and functional comparisons were performed under standardized 1 Hz field stimulation—after a brief (<5 min) time period on the heating pad used for microscopy, *μ*HTs of both genotypes beat at spontaneous rates <1 Hz, allowing us to efficiently drive paced contractions at this common frequency.

Representative cytoplasmic Ca^2+^ traces from *μ*HT working against stiff posts demonstrated that increased cFB density was associated with increased background-corrected transient amplitude (ΔF/F_0_) in both genotypes, with the largest amplitude increases observed at higher cFB fractions [Fig. 3(a)]. Because we held cell density in each *μ*HT constant, tissues made with 25% cFB have fewer iPSC-CMs than *μ*HTs made with lower cFB fractions. This suggests that our observed upregulation in ΔF/F_0_ slightly underestimates per-cardiomyocyte Ca^2+^ intake when the cFB fraction is increased.

Heatmap visualization of kinetic parameters averaged over many *μ*HTs further confirmed that Ca^2+^ calcium handling is strongly shaped by mechanical afterload and stromal cell density [Fig. 3(b)]. Overall, calcium intake increased in proportion to cFB density across both post stiffnesses and in both genotypes. Likewise, Ca^2+^ decay metrics changed with cFB fraction under both soft and stiff loading in control and MYBPC3fs tissues, indicating that fibroblast enrichment is a consistent driver of calcium transient amplitude and decay kinetics across conditions. On soft posts, genotype-based differences in calcium transient kinetics were most apparent in the presence of cFB. Specifically, when working against soft posts, the MYBPC3fs *μ*HT tended to show shorter calcium upstroke duration (UPD) and faster decay, particularly when fibroblasts were present [Figs. 3(c) and 3(d)]. At the same time, calcium intake (peak ΔF/F_0_) remained broadly similar between genotypes [Fig. 3(e)]. When working against stiff posts, calcium kinetics showed clearer genotype-driven differences, but the direction of genotype-driven changes depended on cFB fraction. MYBPC3fs tissues had consistently shorter Ca^2+^ UPD than controls at 0%, 5%, and 25% cFB [Fig. 3(f)]. Time to decay of the Ca^2+^ to 75% from peak (τ_75_) was similar between genotypes at 0% and 5% cFB, but was shorter in MYBPC3fs at 25% cFB [Fig. 3(g)]. Peak Ca^2+^ intake (denoted as background-corrected amplitude, ΔF/F_0_) showed a fibroblast-dependent switch: it was lower in MYBPC3fs at 0% cFB, unchanged at 5% cFB, and higher in MYBPC3fs at 25% cFB [Fig. 3(h)]. Collectively, these findings indicate that MYBPC3fs calcium handling is tuned by mechanical afterload and fibroblast density, paralleling the load- and cFB-amplified hypercontractility observed in force measurements.

### Fibroblasts enhance myofibrillar alignment in isogenic control but not in HCM-mutant *μ*HT

**FIG. 4.**
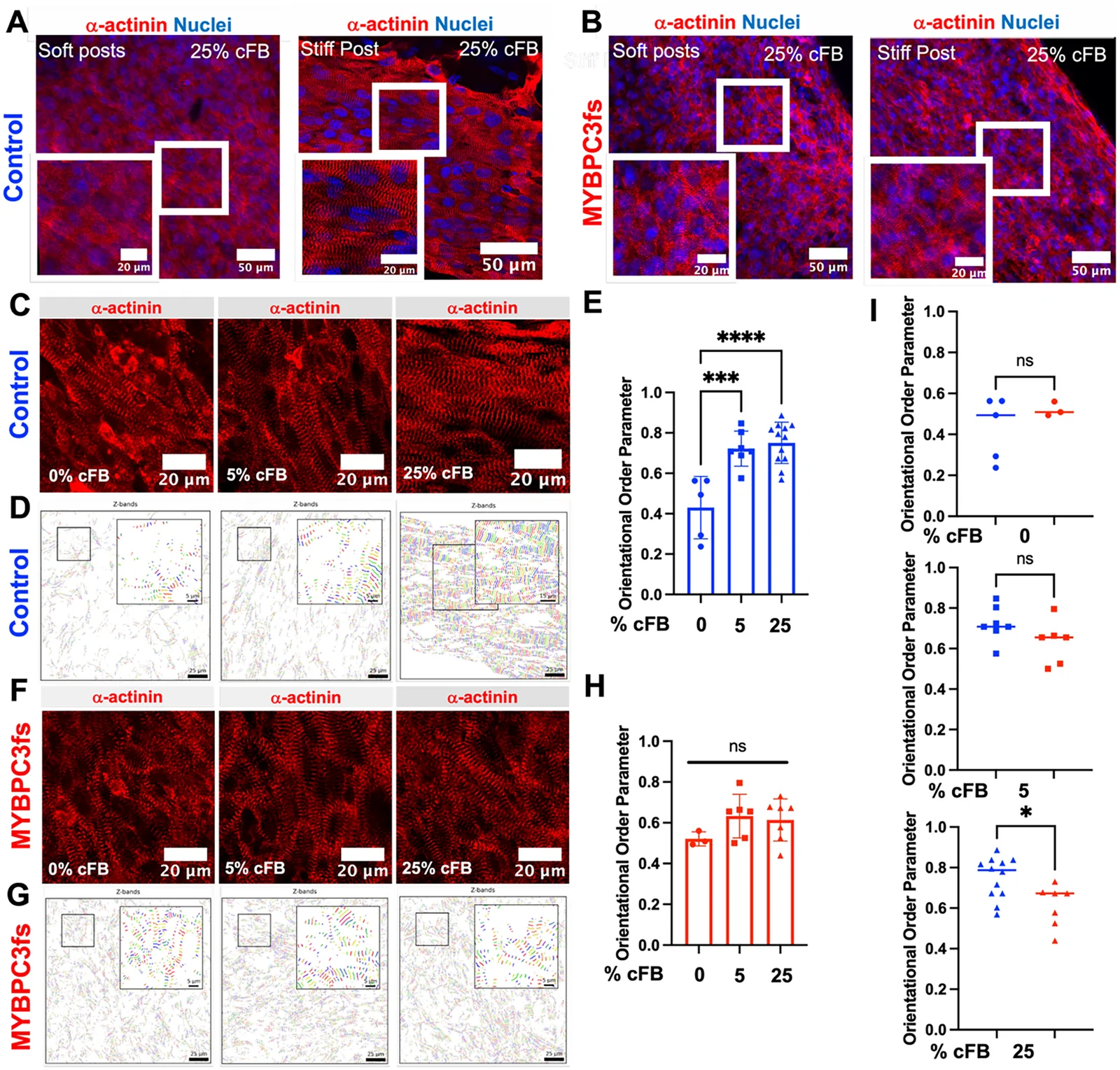
MYBPC3fs-deficient tissues show early structural defects in sarcomeres with increasing mechanical loading compared to controls. Representative immunostaining confocal micrographs of (a) isogenic control tissues containing 25% cFB on soft posts (top-left) and on stiff posts (top-right), and (b) mutant tissues containing 25% cFB on soft posts (bottom-left) and on stiff posts (bottom-right), stained for cardiomyocytes (α-actinin, red), and nuclei (Hoechst, blue). (c) Representative confocal micrographs of isogenic control tissues containing 0%, 5%, and 25% cFB, stained for cardiomyocytes (α-actinin, red) on stiff posts. (d) Z-bands of isogenic control tissues containing 0%, 5%, and 25% cFB on stiff posts. (e) Sarcomere organization in isogenic control tissues increased cFB compositions on stiff posts. (f) Representative confocal micrographs of HCM mutant tissues containing 0%, 5%, and 25% cFB, stained for cardiomyocytes (α-actinin, red) on stiff posts. (g) Z-bands of HCM mutant tissues containing 0%, 5%, and 25% cFB on stiff posts. (h) Sarcomere organization in isogenic control tissues with increased cFB compositions on stiff posts. (i) Comparison of sarcomere organization between control and mutant tissues across fibroblast fractions. N values represent ≥3 independent batches. Error bar: *SD*. ∗, ∗∗∗, and ∗∗∗∗ indicate *p* value less than 0.05, 0.001, and 0.0001, respectively.

Given the strong dependence of contractile performance on fibroblast composition and afterload, we next asked whether the functional divergence in *μ*HTs made from control vs MYBPC3fs iPSC-CM reflected differences in cellular distribution and/or cell-level structural organization. In tissues containing 25% cFB, Z-disk visualization with sarcomeric α-actinin immunostaining revealed that working against stiff posts improved overall sarcomeric registration and alignment in both genotypes relative to soft posts [Figs. 4(a) and 4(b)], consistent with afterload-dependent structural maturation at the tissue scale seen in prior work.^24,79^ Grossly, *μ*HTs made from either genotype had similar distribution and fraction of cardiomyocytes (e.g., area staining positive for α-actinin).

We therefore next assessed whether cFB enrichment differentially influenced myofibrillar organization (orientational order parameter, OOP^80^) between genotypes. In isogenic control *μ*HT working against stiff posts, increasing cFB fraction was associated with greater myofibrillar alignment, with the largest increase observed at 25% added cFB [Figs. 4(c)–4(e)]. In contrast, MYBPC3fs tissues showed non-significant gains in OOP as input cFB density was increased [Figs. 4(f)–4(h)]. This suggested impaired structural adaptation despite robust contraction (Fig. 2). Direct genotype-based comparisons demonstrated the largest divergence in cardiomyocyte organization in *μ*HTs made from 25% cFB [Fig. 4(i)]. This mirrored our observation of spatially disorganized contraction dynamics on the whole *μ*HT level [Fig. 2(f)]. These results establish a functional-structural dissociation in the MYBPC3fs *μ*HT: fibroblast enrichment and afterload can amplify force, yet structural remodeling and alignment fail to improve proportionally in mutant tissues, consistent with “hypercontractile but disorganized” myocardium at the tissue scale.

### TGF-β imposes a dominant pro-fibrotic functional shift characterized by depressed active output, reduced Ca^2+^ intake, increased passive load, and heightened mutant structural changes

HCM progression in patients and animal models commonly involves fibroblast activation and profibrotic signaling.^32,50^ Our preceding results (Figs. 1–4) are consistent with these historical observations, indicating that MYBPC3fs phenotypes are amplified by fibroblast enrichment and mechanical load. As both fibroblast density and mechanical load engage pro-fibrotic remodeling pathways, we next asked whether direct engagement of tissue-wide TGF-β signaling, one of the key pathways underlying fibrosis, is sufficient to drive *μ*HT dysfunction and remodeling.^32,81^ We also explored the possibility that MYBPC3fs tissues exhibit heightened susceptibility to this pro-fibrotic stimulus.

**FIG. 5.**
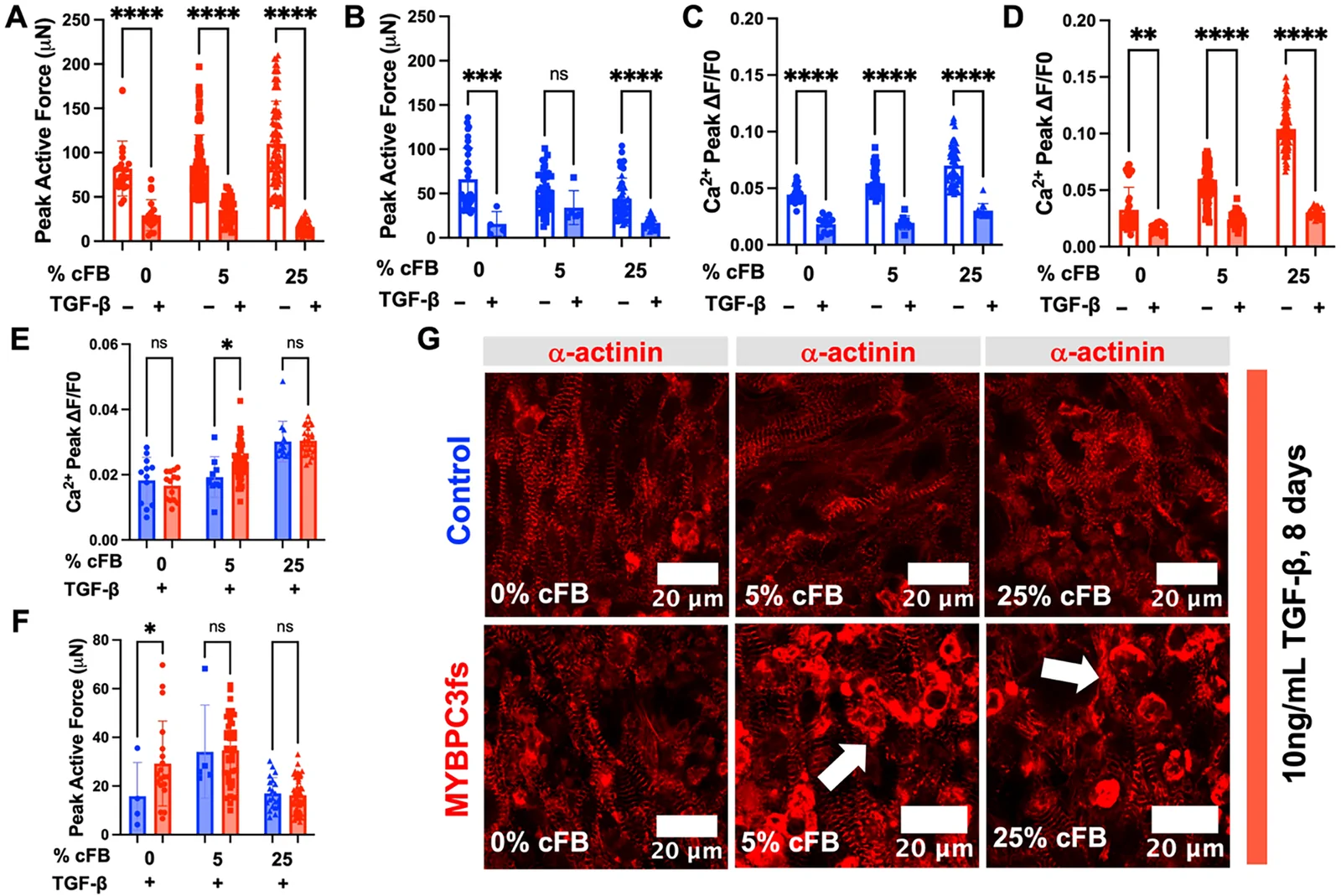
TGF-β provokes contractile defects and calcium mishandling in tissues. (a) Active force of isogenic control tissues on stiff posts. (b) Active force of mutant tissues on stiff posts. (c) Background-corrected peak Ca^2+^ intensity of control tissues on stiff posts. (d) Background-corrected peak Ca^2+^ intensity of mutant tissues on stiff posts. (e) Active force of control and mutant tissues on stiff posts with TGF-β exposure. (f) Background-corrected peak Ca^2+^ intensity of control and mutant tissues on stiff posts with TGF-β exposure. (g) Representative immunostaining images of control and mutant tissues after exposure to TGF-β, stained for cardiomyocytes (α-actinin, red) on stiff posts. *μ*HTs were treated with 10 ng/ml TGF-β for 8 days. N values represent ≥3 independent batches. Error bar: *SD*. ∗, ∗∗, and ∗∗∗∗ indicate *p* value less than 0.05, 0.01, and 0.0001, respectively.

To directly test how pro-fibrotic signaling reshapes function, we exposed *μ*HT working against stiff posts to 10 ng/ml TGF-β for 8 days and assessed contractility, Ca^2+^ dynamics, and structural organization. With chronic TGF-β exposure, peak active force was broadly reduced [Figs. 5(a) and 5(b)]. In MYBPC3fs tissues, the impact of TGF-β on contractility was stronger than in isogenic controls, potentially indicating broader functional sensitivity to profibrotic stimulation. Peak Ca^2+^ intake was significantly reduced by TGF-β exposure across all fibroblast fractions in both genotypes [Figs. 5(c) and 5(d)], consistent with a reported ability of TGF-β signaling to suppress excitation–contraction coupling in cardiomyocytes.^82^

Overall, in assessing the impact of iPSC-CM genotype on the severity of response to chronic TGF-β treatment, MYBPC3fs tissues retained higher peak active force than controls, specifically at 0% cFB [Fig. 5(e)], whereas Ca^2+^ intake differences were smaller [Fig. 5(f)]. Notably, TGF-β altered how fibroblast content related to force output: whereas fibroblast enrichment under baseline conditions could coincide with stronger force [and amplified mutant hypercontractility under load; Fig. 2(c), right], in the presence of TGF-β, higher cFB fractions were associated with weaker active force [Fig. 5(e)]. This suggests a shift toward a tissue state in which increasing stromal content and/or the content of stromal cell-derived ECM no longer supports and can instead limit active force generation.

In addition to suppressing maximum contraction force and peak Ca^2+^ intake, exogenous TGF-β exposure further supported a shift toward impaired mechanical performance and coordination. In the MYBPC3fs *μ*HT working against stiff posts, TGF-β treatment slowed the contraction kinetics when the cFB fraction was 25% [supplementary material, Fig. 4(a)]. Notably, although at baseline the MYBPC3fs *μ*HT had faster relaxation kinetics than the isogenic control tissues, relaxation kinetics were slowed markedly, to the point that relaxation kinetics were not significantly different between the mutant and control *μ*HTs, when tissues with a high cFB fraction were treated with TGF-β [supplementary material, Fig. 4(b)]. In *μ*HTs working against stiff posts, calculated mechanical energy consumption decreased across all the conditions [supplementary material, Fig. 4(c)]. Directional uniformity of motion was suppressed in both genotypes with TGF-β exposure, though this effect was more severe with 25% cFB [supplementary material, Fig. 4(d)]. Moreover, peak strain under TGF-β was higher in MYBPC3fs tissues at 5% and 25% cFB but not at 0% [supplementary material, Fig. 4(e)].

**FIG. 6.**
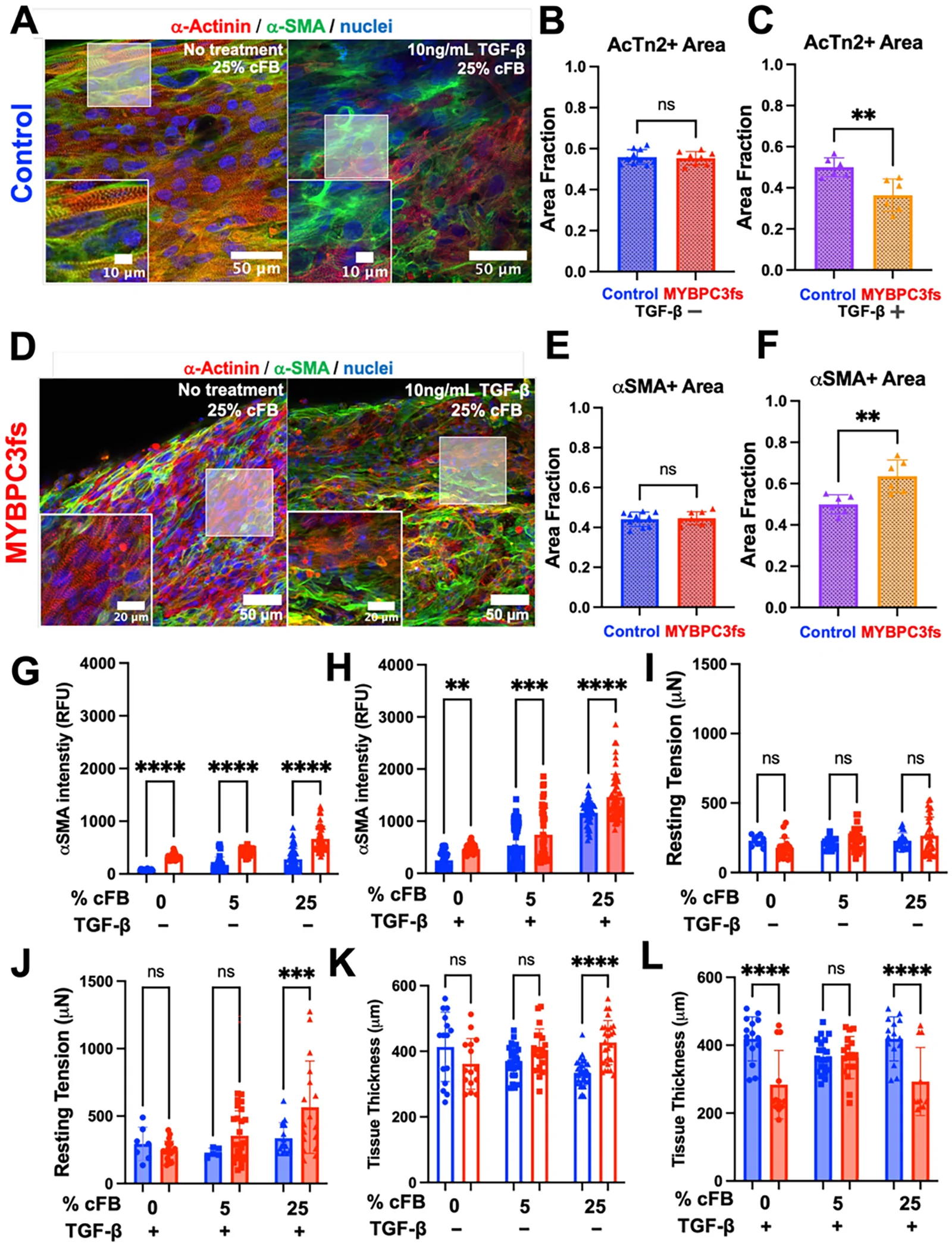
TGF-β provokes tissue-level fibrosis in *μ*HTs. (a) Representative confocal micrographs of isogenic control tissues containing 25% cFB stained for myofibroblasts (α-SMA, green), cardiomyocytes (α-actinin, red), and nuclei (blue). (b) Percentage of α-actinin-positive areas in isogenic control and mutant tissues containing 25% cFB on stiff posts. (c) Percentage of α-actinin-positive areas in isogenic control and mutant tissues containing 25% cFB on stiff posts with TGF-β treatment. (d) Representative confocal micrographs of mutant tissues containing 25% cFB stained for myofibroblasts (α-SMA, green), cardiomyocytes (α-actinin, red), and nuclei (blue). (e) Percentage of α-SMA-positive areas in isogenic control and mutant tissues containing 25% cFB on stiff posts. (f) Percentage of α-SMA-positive areas in isogenic control and mutant tissues containing 25% cFB on stiff posts with TGF-β treatment. (g) α-SMA intensity in isogenic control and mutant tissues associated without TGF-β treatment on stiff posts. (h) α-SMA intensity in isogenic control and mutant tissues associated with TGF-β treatment on stiff posts. (i) Resting tension of isogenic control and mutant tissues associated without TGF-β treatment. (j) Resting tension of isogenic control and mutant tissues associated with TGF-β treatment. (k) Tissue thickness of isogenic control and mutant tissues associated without TGF-β treatment. (l) Tissue thickness of isogenic control and mutant tissues associated with TGF-β treatment. N values represent ≥3 independent batches. Error bar: *SD*. ∗∗, ∗∗∗, and ∗∗∗∗ indicate *p* value less than 0.01, 0.001, and 0.0001, respectively.

TGF-β treatment was also associated with condition-dependent differences in tissue geometry. MYBPC3fs tissues were significantly thinner than controls at 0% and 25% added cFB, whereas no significant genotype difference in thickness was detected at 5% added cFB [Fig. 6(l)]. Despite these geometric differences, estimated active stress did not differ significantly between genotypes at any added-cFB fraction following TGF-β treatment [supplementart material, Fig. 4(f)]. Thus, TGF-β exposure was associated with genotype- and cFB-condition-dependent changes in tissue geometry, but the observed total-force differences did not correspond to significantly greater estimated active stress in the MYBPC3fs *μ*HT. This suggested that profibrotic remodeling modifies tissue geometry and internal load distribution rather than simply reducing cardiomyocyte contractility.

Direct Z-disk visualization indicated that TGF-β exposure was associated with increased sarcomere disorganization, particularly in the MYBPC3fs *μ*HT [Fig. 5(g)]. Control tissues maintained regions of recognizable sarcomeric striation across fibroblast fractions, whereas MYBPC3fs tissues displayed more heterogeneous α-actinin patterns with prominent disarray, particularly in fibroblast-containing tissues [Fig. 5(g)]. TGF-β treatment also triggered the presence of large, poorly organized regions of α-actinin, which may reflect aggregation of this protein. In prior work, we have observed similar α-actinin aggregation when cardiomyocyte proteostasis was disrupted.^73^ Collectively, these findings suggest that while TGF-β provokes a poorly contractile state regardless of iPSC-CM genotype, the impact of fibroblast activation is more severe in *μ*HTs made from iPSC-CMs harboring an HCM-associated genotype.

### TGF-β preferentially promotes fibroblast activation and loss of cardiomyocyte structural area in MYBPC3fs tissues, paralleling increased passive tension

Given the severity of iPSC-CM Z-disk deterioration within the MYBPC3fs *μ*HT after chronic TGF-β exposure, together with a decline in iPSC-CM Ca^2+^ intake and overall contractile function (Fig. 5), we next investigated the potential that replacement fibrosis occurs under these conditions. To address this possibility, we quantified the overall area of each confocal cross section Of the *μ*HT occupied by cardiomyocytes (α-actinin-positive area fraction) and activated fibroblasts (intensity of α-SMA stain) as surrogates for the percentage of each cell type within the tissue [Figs. 6(a)–6(f)]. At baseline (no TGF-β exposure), α-actinin+ area did not differ significantly as a function of iPSC-CM genotype, even in the presence of 25% cFB [Fig. 6(b)]. Likewise, α-SMA+ area was also similar across genotypes without TGF-β exposure [Fig. 6(e)]. Following TGF-β exposure, however, MYBPC3fs tissues exhibited a significant reduction in cardiomyocytes (α-actinin positive area) relative to controls [Fig. 6(c)]. This was accompanied by a significant increase in α-SMA+ area [Fig. 6(f)], consistent with a shift toward a more activated stromal state and with replacement of cardiomyocytes by cFB.

Interestingly, regardless of whether *μ*HTs were supplemented with exogenous TGF-β or not, the average intensity of α-SMA staining within areas manually identified as being α-SMA positive was significantly higher in MYBPC3fs tissues than in isogenic controls [Fig. 6(g)]. TGF-β supplementation exacerbated this genotype-driven increase in α-SMA expression within cFB [Fig. 6(h)]. Cell-level changes in α-SMA expression levels were accompanied by tissue-level increases in resting (diastolic) tension. Without TGF-β, resting tension was similar between control and MYBPC3fs tissues, but MYBPC3fs showed slightly higher resting tension with cFB content, previously used as a surrogate for fibroblast activation state^77^ [Fig. 6(i)]. TGF-β had little effect on resting tension when fibroblasts were absent but increased passive load. Specifically, MYBPC3fs showed an increase, but it was not statistically significant at 5% cFB and became strongly elevated at 25% cFB [Fig. 6(j)], demonstrating heightened passive mechanical changes in mutant tissues under profibrotic stimulation with higher cFB composition. MYBPC3fs tissues were also thinner than controls in the TGF-β-treated 25% added-cFB condition [Fig. 6(l)], indicating that the increase in resting tension was not attributable to greater tissue thickness.

### Alternative cFB sources support similar baseline functionalities in control *μ*HTs

To determine whether our observations on cardiomyocyte–cFB crosstalk might be extended to isogenic cFB in future work, we performed a proof-of-concept study using isogenic control iPSC-derived cFBs. iPSC-cFBs exhibited fibroblast-like morphology and vimentin expression and could be used to form *μ*HTs when combined with control iPSC-CMs [supplementary material, Figs. 6(a) and 6(b)].

Interestingly, we observed no statistically significant differences in *μ*HT resting tension, peak Ca^2+^ transient amplitude, or Ca^2+^ upstroke duration [supplementary material, Figs. 6(c)–6(e)] when isogenic iPSC-cFBs were used to form the tissues. These findings demonstrate the feasibility of incorporating iPSC-cFBs into the *μ*HT platform and suggest that the selected baseline measurements were not unique to the primary cFB preparation under this experimental condition.

## DISCUSSION

In this study, we leveraged a human iPSC-derived micro-heart tissue (*μ*HT) system in which we independently modulated mechanical afterload (PDMS post-bending stiffness) and stromal composition (0%–25% cFB). We used this platform technology to interrogate how an HCM-relevant *MYBPC3* truncating variant acted to sensitize cardiomyocytes to the presence of these two distinct cues, leading to changes in contractile function, excitation–contraction coupling, and tissue remodeling. Across our dataset, three mechanistic themes emerge: (1) afterload and cFB act as “phenotype amplifiers” that convert subtle genotype differences into robust hypercontractility that is associated with excessive energy consumption; (2) Ca^2+^ remodeling in MYBPC3fs cardiomyocytes is strongly dependent on the combination of afterload and cFB fractions; and (3) mutant tissues exhibit uncoupling between structure and function, with greater force and stress without commensurate improvements in sarcomere order, particularly under fibroblast-rich and pro-fibrotic conditions. These findings align with the clinical complexity of hypertrophic cardiomyopathy (HCM), where genotype, loading history, and fibrosis interact to determine disease trajectory and arrhythmic risk.^4,32,36,83–85^

The first major theme that emerges from our findings is that afterload amplifies MYBPC3fs-driven hypercontractility, converting a modest genotype difference on compliant (soft) posts into a robust force phenotype on stiff posts, particularly when cFBs are present (Fig. 2). This “load amplification” indicates that the effects of sarcomeric variants can appear muted when mechanical demand is low but become prominent when the contractile system must generate higher stress. This is in line with prior *in vivo* studies, in which *MYBPC3* mutant mice did not show pathologic hypertrophy at baseline, but had an exaggerated response to afterload caused by transaortic constriction surgery.^30^ Mechanistically, diminished *MYBPC3* expression triggered by the loss-of-function truncating variant would be expected to alter thick-filament regulation and cross-bridge availability, biasing the myocardium toward higher active stress generation, especially when working against high afterload. This interaction between genetic perturbation and mechanical context is highly consistent with our team's prior work with engineered micro-heart muscles made from iPSCs harboring a similar loss-of-function *MYBPC3* variant. In that prior work, the mutant tissues were hypercontractile, yet exhibited reduced tolerance to increased mechanical stiffness, consistent with a “mechanically fragile” hypercontractile state.^20^ This “fragile state” is defined by a mutant myocardium that can generate greater active stress, with the caveat that this elevated contractile output is more strongly shaped and potentially destabilized by increased mechanical demand. In a similar vein, Ma and colleagues^28^ used engineered cardiac micro-tissues with environmental stressors and reported that MYBPC3 deficiency synergizes with mechanical stress to exacerbate dysfunction and calcium abnormalities, emphasizing that stress context can be necessary to elicit clinically relevant phenotypes. In another study with similar findings, Ramachandran *et al.* used a *μ*HT model with varied afterload to demonstrate that a different pathogenic *MYBPC3* truncating variant exhibited hypercontractility when challenged with high afterload. Altogether, our current study and these prior findings suggest a model in which stress magnifies a hypercontractile, pathologic state in MYBPC3-linked HCM.^20,24,28,86^

In addition to supporting the concept that afterload acts as a critical trigger for HCM pathogenesis, our work is also consistent with prior findings suggesting that fibroblasts are not merely “background” cells in the myocardium. Instead, fibroblasts act as amplifiers of the HCM phenotype, most clearly evident in the stronger genotype-driven separation of contractility and structural remodeling at intermediate-to-high cFB fractions (5%–25%) and higher afterload (Figs. 2, 4, and 5). This behavior is consistent with prior literature showing that fibroblasts shape myocardial mechanics and disease progression through ECM remodeling, paracrine signaling, and modulation of cardiomyocyte mechanotransduction, effectively changing the “operating point” of cardiomyocytes. Even when intrinsic myofilament pathogenic variants are the initiating driver of HCM,^2–4,18^ fibroblast pathology plays a clear role in this disease.^56,59^ The present study and prior work^23^ show that this crosstalk can be modeled in cell culture. However, several genotype-associated differences were detected predominantly under the 25% added-cFB condition. The present design therefore does not establish whether these responses are linear, monotonic, nonlinear, or threshold dependent. Additional intermediate cFB fractions, such as 10% or 15%, will be required to define the shape of the response.

Given that fibroblasts and afterload reshape the mechanical and signaling environment experienced by cardiomyocytes, we also examined whether MYBPC3fs calcium handling also varies as a function of this mechanical–stromal context. Calcium dynamics in MYBPC3fs tissues argue against a simple, genotype-invariant calcium-handling phenotype (e.g., “always higher Ca^2+^ amplitude” or “always slower decay”). Instead, MYBPC3fs calcium dynamics re-parameterize with afterload and fibroblast fraction, with shifts in upstroke/decay kinetics and altered peak ΔF/F_0_ emerging under stiff loading with higher cFB fractions [Fig. 2(b)]. This behavior aligns with prior studies^20,24^ showing that mechanically challenging environments can unmask excitation–contraction coupling abnormalities. For example, in our prior study,^24^ using micro-heart muscle arrays containing iPSC-CMs with a *MYBPC3*^+/−^ variant, we found that afterload on the tissues increased pathological calcium intake in the mutant tissues. These results suggest that pathological remodeling of calcium handling in HCM cardiomyocytes evolves in a load- and stroma-sensitive manner. This is consistent with prior reports that sarcomeric variants can alter force generation and energetic cost without requiring proportional increases in Ca^2+^ transient amplitude.^24^ Mechanistically, this is compatible with the broader idea that sarcomeric HCM-linked variants can increase contractile output through myofilament-level changes (cross-bridge kinetics and energetic cost per unit force) without requiring proportionate increases in Ca^2+^ transient amplitude.^87–91^ In that scenario, higher force at similar or lower Ca^2+^ signal reflects a shift in excitation–contraction coupling “gain” (force per unit Ca^2+^) rather than a more efficient cardiac cycle. Indeed, apparent increases in force-per-Ca^2+^ could coexist with unchanged or worsened energetic cost if greater cross-bridge cycling, faster detachment, and/or tissue-scale mechanical inefficiency (e.g., strain heterogeneity and sarcomere disarray) increase ATP demand, particularly under high afterload.^86,88,90–93^

A second major insight from our study is the apparent structure/function uncoupling in the mutant *μ*HT. Control iPSC-CMs exhibited fibroblast-dependent improvements in myofibrillar alignment within *μ*HT working against high afterload (Fig. 4). In contrast, MYBPC3fs tissues showed blunted alignment gains across fibroblast fractions despite stronger contractile force production. In controls, increasing cFB fraction improved cardiomyocyte and sarcomere alignment [Fig. 4(c)], consistent with the concept that stromal cells provide structural guidance and paracrine cues that support maturation and alignment in engineered myocardium.^94–96^ This is broadly consistent with tissue engineering literature showing that microenvironmental patterning/organization cues can promote cardiomyocyte alignment and function.^94,97–99^ In MYBPC3fs tissues, however, iPSC-CMs failed to show improved alignment in the presence of cFB [Figs. 4(f)–4(h)]. This was concurrent with spatially disorganized tissue contraction that became statistically significant when afterload and cFB density were high [Fig. 4(i)]. One cohesive interpretation is that *MYBPC3* truncation shifts cardiomyocytes toward a mechanically overdriven baseline state, especially with high afterload.^22,24,86^ In that state, elevated internal stress/strain heterogeneity^92,100,101^ can impede ordered sarcomere remodeling even if fibroblasts provide structural alignment cues.^95^ This “hypercontractile but disorganized” phenotype clearly aligns with the hallmark features of human/clinical HCM, specifically the hypercontractility observed on functional imaging studies, in conjunction with the myocyte disarray seen on pathologic specimens. Our data are also consistent with prior work by Cohn and colleagues^22^ that reported that myofibrillar disarray can occur in parallel with hypercontractile function.

Direct measurement of tissue thickness refined the interpretation of the force data. In untreated stiff-post tissues containing 25% added cFB, MYBPC3fs tissues were significantly thicker than controls, and the genotype difference in total active force was no longer apparent after normalization to estimated cross-sectional area [Figs. 2(c), 6(k), and supplementary material, Fig. 3(f), right]. This indicates that the high-force phenotype in this condition is influenced, at least in part, by tissue geometry and should not be interpreted as evidence of increased intrinsic active stress.

The geometric response was not uniform across conditions. Following TGF-β treatment, MYBPC3fs tissues were thinner than controls at 0% and 25% added cFB, yet estimated active stress did not differ significantly between genotypes. Together, these observations show that genotype, cFB content, and TGF-β exposure modify tissue dimensions in a condition-dependent manner. They do not suggest a generalized increase in MYBPC3fs tissue size or tissue-level hypertrophy.

Notably, we did not observe a robust defect in diastolic relaxation in the MYBPC3fs *μ*HT, even though impaired mechanical relaxation is often expected in clinical HCM.^4,102^ This finding is consistent with our prior micro-heart muscle array work, where genotype-stiffness interactions produced clear hypercontractility without a strong relaxation deficit.^20^ Importantly, other mechanistic HCM studies have reported phenotypes consistent with diastolic dysfunction, although the direction of the change varies across model systems and readouts. For example, in a mouse *MYBPC3* mutant EHT model, Stöhr *et al.* reported altered twitch kinetics with faster relaxation.^103^ In contrast, dysregulated myosin regulation and impaired (longer) diastolic relaxation were demonstrated in *MYBPC3* mutant mouse cardiomyocytes.^8^ Moreover, slower relaxation has also been described in several iPSC-CM HCM models carrying pathogenic *MYH*7 or *MYBPC3* variants.^22,28,104^ These disparate observations suggest that diastolic/relaxation dysfunction may not be solely driven by HCM-associated variants, but rather may also be an emergent phenotype that depends on the mechanical regime, culture duration, maturation state, and structure–function alignment of the tissue. In our platform, tissues exhibited a “compensated hypercontractile” state in which myofilament activation and force generation are sensitized by afterload/stromal context, but the assay conditions used in our study may not cross a threshold sufficient to produce a consistent relaxation defect. Interestingly, we did observe that the HCM *μ*HT, which had faster relaxation kinetics than controls at baseline, showed some slowing of their diastolic relaxation in the presence of activated fibroblasts, highlighting a potential role for these stromal cells in the diastolic pathology of HCM.

Profibrotic signaling has been proposed to be an important driver of both the development of HCM and adverse remodeling in HCM, with TGF-β widely recognized as a major pathway promoting fibroblast activation and myofibroblast differentiation.^50,62^ Indeed, when applied prophylactically, neutralizing antibodies against TGF-β slowed the development of HCM pathology in a mouse model of this disease.^32^ Myofibroblast activation promotes ECM remodeling and changes in myocardial stiffness and function.^54,55^ TGF-β suppressed active force and Ca^2+^ intake across *μ*HTs of both genotypes [Figs. 4(a)–4(d)]. Suppressed contractility and Ca^2+^ intake were accompanied by worsened Z-disk organization and increased passive tension. Weakened contractility [Figs. 5(a) and 5(b)] is likely a result of direct effects of TGF-β on the cardiomyocytes themselves^68,81^ and heightened fibroblast activation,^105^ which would lead to maladaptive, fibrotic remodeling of the tissue. On the cellular level, we further observed that an increase in α-SMA-positive area reduced the presence of α-actinin-positive cardiomyocytes when TGF-β was added, suggesting some replacement fibrosis. These findings are overall consistent with recent work by Ewoldt and colleagues,^23^ in which those authors demonstrated that HCM-associated variants can activate stromal cell proliferation and ECM deposition via EGFR-mediated paracrine signaling. Notably, however, these authors observed baseline activation of cFBs by iPSC-CMs with HCM-linked variants, whereas in the present study, we found that cFBs in *μ*HTs made from HCM cardiomyocytes were more prone to activation given exogenous TGF-β, and that the baseline level of activation in the absence of TGF-β was low. Despite this difference, our findings and the prior study are both consistent with the broader concept that HCM-associated variant-driven stress states can lower the threshold for stromal activation and remodeling.

A shared primary human ventricular cFB source was used across control and MYBPC3fs tissues so that cardiomyocyte genotype could be varied within a common stromal background. This choice was justified by the general acknowledgment that MYBPC3 transcript expression is cardiomyocyte-restricted. As an initial assessment of fibroblast-source dependence, we also compared resting tension in control tissues containing 25% primary cFBs or 25% iPSC-derived cFBs [supplementary material, Fig. 6(c)]. No significant difference was detected under untreated stiff-post conditions, indicating that baseline resting tension in this specific setting was not unique to the primary cFB preparation and demonstrating the feasibility of incorporating iPSC-derived cFBs into the *μ*HT platform. In future studies, it will be of interest to leverage iPSC-cFBs to explore the role of cFB genomic background on heart muscle physiology. We note that primary adult and iPSC-derived cFBs may differ in developmental state, culture history, activation threshold, matrix production, and responses to TGF-β. In addition, human HCM myocardium contains disease-associated activated fibroblast states that may not be represented by either a non-diseased primary cFB donor or iPSC-cFBs.^106,107^

We acknowledge several limitations of our study. First, iPSC-derived cardiomyocytes exhibit incomplete maturation, which can influence baseline kinetics and stress responses.^108^ Second, while the primary human fibroblasts are widely used in cardiac tissues, they are neither genetically nor developmentally age-matched to the cardiomyocytes, which may affect activation thresholds and ECM output. Although resting tension was similar between control *μ*HTs containing primary or iPSC-derived cFBs under one condition, this limited comparison does not establish source equivalence across other outcomes. Third, the 0%, 5%, and 25% conditions represent targeted added-cFB input fractions rather than directly measured final tissue compositions. These three conditions do not define whether cFB-associated responses are graded or threshold-dependent and should not be mapped directly onto stages of clinical HCM. Fourth, tissue thickness was measured only in representative stiff-post tissues and averaged within each experimental condition. Cross-sectional area was therefore estimated using individual tissue widths and condition-specific mean thicknesses rather than paired three-dimensional measurements from every tissue. Revised geometry-normalized stress analyses were consequently restricted to stiff-post conditions. Fifth, TGF-β is a potent, ubiquitous stimulus that acts on both cardiomyocytes and fibroblasts;^81,109^ thus, the use of exogenous TGF-β as a chemical stimulant for fibrosis may limit our ability to isolate and recapitulate endogenous cardiomyocyte–fibroblast crosstalk. Extracellular-matrix deposition and remodeling were also not directly quantified. Accordingly, changes in α-SMA expression, tissue organization, and resting tension should be interpreted as markers of a tissue-level profibrotic response rather than direct measurements of fibrosis burden or fibroblast-specific causality.

Having established this *in vitro* model system to study cardiomyocyte-cardiac fibroblast crosstalk in HCM pathogenesis, future studies are aimed at directly quantifying the dynamics of ECM remodeling and resultant changes in passive tissue mechanics and incorporating intermediate added-cFB fractions, additional primary cFB donors, and genetically matched control and MYBPC3fs iPSC-derived cFBs. A factorial design in which cardiomyocyte and fibroblast genotypes are varied independently could help distinguish cardiomyocyte-autonomous, fibroblast-autonomous, and reciprocal signaling effects. Reduction of *μ*HT size and/or use of simplified, 2D or semi-2D screening platforms may aid such studies. Future studies could also use fibroblast-biased profibrotic cues, such as placental growth factor^110^ or connective tissue growth factor.^111^ More definitive mechanistic separation is likely to require genetic approaches that allow true fibroblast-restricted signaling, such as selective activation of TGF-β receptor–SMAD2/3 signaling^112^ in cFBs, followed by assessment of downstream cardiomyocyte responses. These studies, together with quantitative measurements of collagen, fibronectin, periostin, matrix organization, and tissue material properties, will help distinguish fibroblast-mediated remodeling from direct cardiomyocyte effects. In future studies, we also plan to determine the mechanistic basis through which the combination of mechanical stress and cFB drives the “hypercontractile but disorganized” phenotype. Finally, it will be interesting in the future to incorporate additional cardiac cell types (endothelial and immune populations)^113–115^ to determine whether and how these cell populations impact fibrosis and cardiomyocyte pathology.

## METHODS

### Human iPSC culture and maintenance

Two iPSC cell lines were used in this study. The isogenic control line was derived from a healthy male with no family history of heart disease (WTC; Coriell repository # GM25256) and was previously edited to express a single genomic copy of the genetic Ca^2+^ indicator GCaMP6f,^116^ enabling optical measurement of cardiomyocyte calcium transients. The WTC-GCaMP6 iPSC were generously provided by Dr. Bruce Conklin (Gladstone Institute of Cardiovascular Disease, San Francisco, CA). To generate the HCM model, a patient-specific pathogenic heterozygous *MYBPC3* frameshift variant, G1206fs (MYBPC3fs),^75^ was engineered into the Control-GCaMP6 background by the Genome Editing and Stem Cell Center at Washington University School of Medicine. Both lines were passaged and maintained in StemFlex media (Life Technologies; A3349401) on plates coated with Geltrex (Life Technologies; A1413302) at a concentration of 18.75 mg/cm^2^. During passaging, iPSCs were dissociated using Gentle Dissociation Reagent (PBS with 1.8 g/l NaCl and 0.5 mM EDTA)^117^ and replated as single cells in StemFlex media supplemented with 10 *μ*M Y27632 (Biogem; 1293823).

### Human iPSC-CM differentiation and purification

Both the isogenic control and MYBPC3fs stem cell lines were differentiated into cardiomyocytes using a small molecule-driven Wnt signaling^118,119^ [Fig. 1(d)]. Briefly, iPSCs were plated at 40 000 cells/cm^2^ on Geltrex-coated plates (37.5 mg/cm^2^) and expanded in StemFlex medium for ∼3 days until confluent (designated Day 0). Differentiation was initiated on Day 0 by switching to RPMI-I media (RPMI1640, Life Technologies, 11875093; 2% B27 without insulin, Life Technologies, A1895601) supplemented with 150 *μ*g/ml ascorbic acid (Fisher Scientific; NC0602549) and 6 *μ*M CHIR99021 (Biogem; 2520691). On Day 2, the media were replaced with RPMI-I containing 150 *μ*g/ml ascorbic acid and 5 *μ*M IWP2 (Biogem; 6866167), followed by RPMI-I with ascorbic acid alone on Day 4. From Day 6 forward, cultures were maintained in RPMI-C (RPMI 1640 with 2% B27 supplement (Life Technologies; 17504044)) with media changes every 2 days. Spontaneous contractions typically emerged between Day 8 and Day 10. To enable downstream tissue fabrication with high cardiomyocyte purity, cells were dissociated on Day 14 using 10× TrypLE Select Enzyme (Gibco; A1217701) on Geltrex-coated plates (37.5 mg/cm^2^) at 285 000 cells/cm^2^ in RPMI-C media supplemented with 20% fetal bovine serum (FBS; Fisher Scientific; FB12999102) and 10 *μ*M Y27632 for 48 h. On Day 16, cells were returned to RPMI-C, and metabolic enrichment was initiated two days later using lactate-based metabolic selection media consisting of glucose-depleted RPMI (Millipore Sigma; R1383) supplemented with 4 mM lactate (Fisher Scientific; AAL1450014), 1% Non-Essential Amino Acids (Fisher Scientific; 11140050), and 1% GlutaMAX (Fisher Scientific; 35050061). Lactate selection media were refreshed every 2 days for a total of 6 days. Beginning on Day 24, cultures were gradually transitioned back to RPMI-C by stepwise dilution of lactate selection media (day 24: 75% lactate/25% RPMI-C; Day 25: 50%/50%; Day 26: 25%/75%). On Day 27, media were fully returned to RPMI-C, and cells were allowed to recover for an additional 3 days. Cardiomyocyte purity was quantified by flow cytometry for cardiac troponin T (cTnT) [Fig. 1(b)]. Purified iPSC-CMs were then used for *μ*HT fabrication.

### Cardiac fibroblast culture

Human primary ventricular cardiac fibroblasts (Lonza; CC-2904) were maintained in fibroblast growth media (Lonza, FBM™ Basal Medium; CC-3131, supplemented with FGM™-2 SingleQuots™ supplements; CC-4126). For dissociation, 0.25% trypsin EDTA (Fisher Scientific; 25200072) was used to generate single-cell suspensions for tissue assembly.

### Generation of iPSC-derived cardiac fibroblasts

Isogenic control iPSCs were differentiated into cardiac fibroblasts through cardiac progenitor and epicardial intermediate stages using the small-molecule-directed protocol described by Zhang *et al.*^120^ Briefly, iPSCs were treated with 6 *μ*M CHIR99021 for 2 days, recovered for 1 day, treated with 5 *μ*M IWR-1 (Millipore Sigma; I0161) for 2 days, and recovered for another day. On Day 6, cardiac progenitor cells were replated at 20 000 cells/cm^2^ in Advanced DMEM (Fisher Scientific; 12491015). On Day 7 or 8, cells were treated with 5 *μ*M CHIR99021 and 2 *μ*M retinoic acid (Millipore Sigma; R2625) for 2 days, followed by 3 days of recovery. The resulting epicardial cells were replated and cultured in fibroblast growth medium (PromoCell; C-23025) containing 10 *μ*M FGF2 (Fisher Scientific; 100-18B) and 10 *μ*M SB431542 (Selleck Chemicals; S1067) for 6 days. Cells generated using this protocol are referred to as iPSC-cFBs. Vimentin staining was used to confirm iPSC-cFB differentiation [supplementary material, Fig. 6(a)].

### Post-based device design, fabrication, and surface modification

The device design and fabrication were performed as previously described.^73,74,121^ Briefly, a double-molding process was used to generate PDMS *μ*HT inserts from a 3D-printed master (printed in FormLabs ClearResin using a Form3 stereolithographic printer), using agar (1.5% wt/v agar, supplemented with 0.2% v/v Triton-X-100) as an intermediary transfer material. Each *μ*HT insert contained 12 microwells, with each microwell containing two posts (1.25 mm height) spaced 1850 *μ*m apart. Stiff posts were cast using pure Sylgard 184 (bending stiffness: 1.96 N/m), whereas soft posts were generated using a Sylgard 184: Sylgard 527 blend (1:4 ratio; bending stiffness: 0.42 N/m). Prior to cell culture studies, the PDMS *μ*HT inserts were attached to polycarbonate cell-culture inserts^122^ and sterilized by autoclaving. To minimize cell adhesion to PDMS and promote tissue self-assembly, sterilized devices were treated with 1% wt/v Pluronic F127 for ≥2 h at room temperature, followed by three rinses in sterile dPBS.

The post-based *μ*HT was selected to overcome limitations observed in prior substrate-adherent engineered tissue formats, in which high cFB densities increased resting tension and promoted tissue delamination [supplementary material, Fig. 2(a)]. In contrast, in the post-based format, tissues remain mechanically stabilized under elevated resting tension, enabling robust formation and maintenance of cFB-enriched *μ*HTs [supplementary material, Fig. 2(b)].

### *μ*HT formation with variable cFB density

Purified iPSC-CMs were dissociated using 10× TrypLE Select Enzyme for 15 min, while cFBs were dissociated using 0.25% trypsin EDTA for 5 min to generate single-cell suspensions. Cells were then combined at defined CM: cFB ratios to produce tissues with targeted fibroblast densities of 0%, 5%, or 25%. For consistency in mixing calculations, the post-selection CM-enriched fraction was assumed to be 100% cardiomyocytes when calculating cell-mixing ratios; accordingly, “5%” and “25%” cFB conditions reflect the targeted cFB proportion at seeding (by total cells), with the remaining cells designated as cardiomyocytes. Cells were suspended at a final density of 2 × 10^7^ cells/ml, in an ECM hydrogel consisting of 1.5 mg/ml collagen I rat tail (Fisher Scientific; A1048301) and 10% v/v Geltrex, and the mixture was neutralized with 1M NaOH to physiological pH. For each microwell, 4 *μ*l of the cell-gel suspension (80 000 cells) was seeded, followed by incubation at 37 °C with 5% CO_2_ for 20 min to allow cross-linking. Wells were then filled with *μ*HT culture media consisting of KnockOut DMEM (Life Technologies; 10829018) supplemented with 20% FBS, 1% GlutaMax, 1% Non-essential Amino Acids, 1% Penicillin/Streptomycin (Fisher Scientific; 15–140–122), 150 *μ*g/ml ascorbic acid, and 10 *μ*M Y27632. Tissue compaction was typically evident within 2 days, and tissues were maintained for a total of 10 days with media changes every 2 days.

### TGF-β treatment as a tissue-level profibrotic challenge

To induce fibrotic remodeling in *μ*HTs, recombinant human TGF-β (PeproTech; AF-100-36E-10UG) was added to the tissue culture media at 10 ng/ml beginning on day 2 of *μ*HT culture and maintained for 8 days. Following treatment, tissues were harvested for fibrosis assessment and functional measurement. Because TGF-β can signal directly in cardiomyocytes and cFBs, this treatment was used as a tissue-level profibrotic challenge rather than a fibroblast-specific activation method.

### High-speed imaging of contractility and calcium transients

Contractility was captured via brightfield videos, and calcium transients were quantified via GCaMP6f fluorescence.^116^ Tissues were imaged on Day 10 of tissue making using an epifluorescence microscope (Nikon Eclipse Ts2R; Tokyo, Japan) equipped with a digital CMOS camera (Hamamatsu ORCA-Flash4.0 V2; Japan), and an Aura II Epifluorescence source with single LED light sources (350, 488, 540, and 647 nm). Recordings were acquired at 100 frames per second for 5 s under the GFP channel. A thermal plate (Tokai Hit; Shizuoka, Japan) was applied and set to a constant 37 °C during imaging. For functional measurements under controlled rhythm, a MyoPacer Field Stimulator (IonOptix) was used to pace *μ*HTs during imaging. Tissues were paced at 1 Hz using 20 ms duration biphasic pulses with a voltage range of 7–20v. Fluorescence was analyzed to extract ΔF/F_0_ (normalized calcium intake), the upstroke time from baseline to peak intensity (UPD), and the time from peak to 30%, 50%, and 75% decay. Quantification was performed using custom MATLAB code available at https://huebschlab.wustl.edu/resources-2/.^123^

### Contractility measurement and force calculation from post deflection

Brightfield videos were recorded at 100 frames per second. Post motion was tracked and analyzed using lab-developed Python code [supplementary material, Fig. 5(a)].

The calibrated effective post-bending stiffness, K, was used to convert changes in the distance between the posts into force. Resting force, representing passive tissue tension, was calculated as

$$F_{\text{rest}}=\left(L_{0}-L_{1}\right)K,$$

where $L_{0}$ is the unloaded post spacing, and $L_{1}$ is the post spacing when the tissue is relaxed [supplementary material, Fig. 5(c)]. Peak active force was calculated as

$$F_{\text{active}}=\left(L_{1}-L_{2}\right)K,$$

where $L_{2}$ is the minimum post spacing at peak contraction [supplementary material, Fig. 5(d)]. Contractile traces were segmented to quantify peak active force, resting tension, contraction time, relaxation time, peak strain, peak active stress, estimated contractile work, and contraction directional uniformity. Peak contractile strain was calculated as the magnitude of the maximum average longitudinal shortening,

$$\varepsilon_{\text{peak}}\%=100\left[-\underset{t}{\text{min}}\left(\frac{1}{N}\sum_{i=1}^{N}\varepsilon_{i}\left(t\right)\right)\right],$$

where $\varepsilon_{i}\left(t\right)$ is the longitudinal strain measured in tissue region i at time t, and N is the number of analyzed regions. The negative sign converts shortening strain into a positive peak value. Spatial displacement vectors were also used to calculate contraction directional uniformity [supplementary material, Fig. 5(b)].

Tissue width was measured from calibrated top-view images [supplementary material, Fig. 5(e)]. Tissue thickness was measured from calibrated side-view images of representative stiff-post tissues in the relaxed state [supplementary material, Fig. 5(f)]. Three measurements were obtained along the central tissue shaft, excluding the enlarged regions adjacent to the posts [supplementary material, Fig. 5(f)]. Measurements were averaged within each tissue and subsequently across tissues within each genotype, added-cFB, and treatment condition to obtain a condition-specific mean thickness.

For stiff-post tissues, cross-sectional area was estimated by approximating the central tissue shaft as an ellipse,^79^

$$A=\frac{\pi wh}{4},$$

where w is the measured width of an individual tissue, and h is the mean thickness for the corresponding experimental condition. Peak active stress was calculated as

$$\sigma_{\text{peak}}=\frac{F_{\text{peak}}}{A}=\frac{4F_{\text{peak}}}{\pi wh}.$$

Because side-view thickness measurements were unavailable for soft-post tissues, cross-sectional area was estimated using the measured width and an assumed circular geometry [supplementary material, Fig. 5(g)],

$$A_{\text{nominal}}=\frac{\pi w^{2}}{4}.$$

Accordingly, stress values for soft-post tissues represent nominal width-based estimates, whereas values for stiff-post tissues incorporate individual tissue width and condition-specific mean thickness.

Estimated contractile work was calculated by integrating force over tissue shortening,

$$E_{\text{contractile}}\approx -\int_{\text{shortening}}F\;dL.$$

### Immunostaining of μHT, imaging, and structural quantification

*μ*HTs were incubated in 100 mM KCl until spontaneous beating ceased, then fixed stepwise at 4 °C with sequential paraformaldehyde concentrations (1%, 2%, 3%, and 4%), with each step performed for at least 1 h. Following fixation, tissues were washed with dPBS three times. Tissues were stained for sarcomeric α-actinin (Millipore Sigma; A7811), Vimentin (Fisher Scientific; MA5-11883), and alpha-smooth muscle actin (Abcam; ab124964). Cell nuclei were stained with Hoechst 33342. Whole tissues were mounted in ProLong Gold (Fisher Scientific; P36930) and imaged on a Fluoview FB1200 confocal microscope (Olympus).

Image-based quantification was performed in ImageJ (NIH). α-actinin+ area, αSMA+ area, and αSMA fluorescence intensity were quantified within regions containing nuclei. For area measurements, binary masks were generated using consistent thresholding parameters: α-actinin+ area was quantified from regions expressing α-actinin, and αSMA+ area was quantified from regions expressing αSMA using the same analysis settings across conditions. For αSMA fluorescence intensity, images were acquired using identical microscope/laser settings (10 ms exposure) for all samples, and mean fluorescence intensity was measured in ImageJ within the nuclear-containing regions of interest. Myofibrillar alignment was assessed from α-actinin images by extracting Z-band orientation and computing the orientational order parameter (OOP) as previously described by Grosberg.^80^

### Comparison of primary and iPSC-derived cFBs in *μ*HT

To evaluate whether fibroblast source influenced baseline passive tissue mechanics, control iPSC-CMs were combined with either primary human ventricular cFBs or iPSC-cFBs at a targeted added-cFB fraction of 25%. Total input-cell number, hydrogel composition, tissue-fabrication procedure, culture duration, and post stiffness were held constant. Untreated tissues were cultured on stiff posts [supplementary material, Fig. 6(b)], and resting tension, ΔF/F_0_, and UPD were quantified on Day 10 using the same analysis as that used for the principal dataset (supplementary material, Figs. 6(c)–6(e)].

### Quantification and statistical analysis

Statistical analyses and data visualization were performed in GraphPad 9.3.1. Data were presented with mean ± standard deviation (SD), and tissue replicates were from at least three independent batches. Normality was assessed using normality and lognormality tests. For two-group comparisons and comparisons among multiple groups with independent variables, a non-parametric Mann–Whitney U test, one-way ANOVA with Dunn's correction, and two-way ANOVA followed by Holm–Šidák *post hoc* test were applied as appropriate. A two-tailed p < 0.05 was considered statistically significant.

## SUPPLEMENTARY MATERIAL

See the supplementary material for the additional experimental results and supplementary figures, as well as a supporting figure that further validates the findings presented in the main text.

## Data Availability

The data that support the findings of this study are available from the corresponding author upon request and within the article and its supplementary material.
